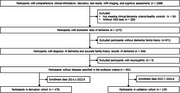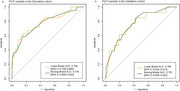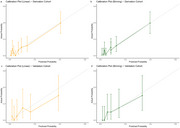# Predictive Accuracy of a Clinical Model for Identifying Pathogenic Gene Carriers in Dementia Patients with a Family History at PUMCH

**DOI:** 10.1002/alz70861_108047

**Published:** 2025-12-23

**Authors:** Jialu Bao, Jing Gao, Chenhui Mao, Liling Dong, Yuyue Qiu, Tianyi Wang, Shanshan Chu, Wei Jin, Bo Li, Yuhan Jiang, Wenjun Wang, Yixuan Huang, Yunfan You, Yuanheng Li, Longze Sha, Feng Feng, Meiqi Wu, Li Huo, Bo Hou

**Affiliations:** ^1^ Peking Union Medical College Hospital, Beijing, Beijing China; ^2^ Institute of Basic Medical Sciences & Neuroscience Center, Chinese Academy of Medical Sciences and Peking Union Medical College, Beijing, Beijing China; ^3^ Peking Union Medical College Hospital, Beijing China

## Abstract

**Background:**

Identifying carriers of pathogenic or likely pathogenic gene variants in dementia is crucial for risk stratification, particularly in individuals with a family history. This study aimed to develop and validate a clinical prediction model using a whole‐exome sequencing‐confirmed cohort.

**Method:**

A total of 601 Chinese dementia patients with a family history were enrolled at Peking Union Medical College Hospital, with 476 in a retrospective derivation cohort and 125 in a temporal validation cohort. Predictive factors included age at onset, apolipoprotein E ε4 status, and family history characteristics. Model performance was evaluated using discrimination and calibration metrics.

**Result:**

In the derivation cohort (median age at onset 66 years), 10.3% had pathogenic or likely pathogenic variants. Early‐onset dementia (odds ratio 2.56, *p* = 0.0098), more than two affected relatives (odds ratio 3.32, *p* = 0.0039), parental history (odds ratio 4.72, *p* = 0.015), and early‐onset family cases (odds ratio 2.61, *p* = 0.0096) were positively associated with variant detection. Apolipoprotein E ε4 carriage was negatively associated (odds ratio 0.36, *p* = 0.0041). The model achieved an AUC of 0.776 (95% CI: 0.701–0.853) in the derivation and 0.781 (95% CI: 0.647–0.914) in the validation cohort (median onset age 58 years), with good calibration.

**Conclusion:**

This model demonstrated strong predictive performance in identifying dementia patients at risk of carrying pathogenic variants, supporting its clinical utility in guiding genetic testing. Further research is needed for external validation and refinement.